# Three-Dimensional Assessment of Upper Airway Volume and Morphology in Patients with Different Sagittal Skeletal Patterns

**DOI:** 10.3390/diagnostics14090903

**Published:** 2024-04-26

**Authors:** Silvia Izabella Pop, Ana Procopciuc, Bianca Arsintescu, Mihai Mițariu, Loredana Mițariu, Radu Vasile Pop, Diana Cerghizan, Kinga Mária Jánosi

**Affiliations:** 1 Faculty of Dental Medicine, George Emil Palade University of Medicine, Pharmacy, Science and Technology of Târgu Mureș, 38 Gh. Marinescu Str., 540139 Târgu Mureș, Romania; 2 Faculty of Dental Medicine, Lucian Balga University, Bd-ul. Victoriei, 550024 Sibiu, Romania; 3 Private Practice, Natural Smile Dental Clinic, 76 Gh Doja Str., 540232 Târgu Mureș, Romania

**Keywords:** skeletal discrepancies, 3D airway analysis, CBCT, orthodontics, malocclusion

## Abstract

Background: The relationship between respiratory function and craniofacial morphology has garnered significant attention due to its implications for upper airway and stomatognathic development. Nasal breathing plays a key role in craniofacial growth and dental positioning. This study investigated upper airway morphology and volume differences among individuals with class I, II, and III skeletal anomalies. Methods: Ninety orthodontic patients’ CBCT scans were analyzed to assess the oropharynx and hypopharynx volumes. Skeletal diagnosis was established based on the cephalometric analysis. Results: A significant volume change in the oropharynx and pharynx was demonstrated when comparing class II with class III anomalies (*p* = 0.0414, *p* = 0.0313). The total volume of the pharynx was increased in class III anomalies. The area of the narrowest part of the pharynx (MIN-CSA) significantly decreased in classes I and II compared to class III (*p* = 0.0289, *p* = 0.0003). Patients with Angle class III anomalies exhibited higher values in the narrowest pharyngeal segment. Gender differences were significant in pharyngeal volumes and morphologies across malocclusion classes. Conclusions: The narrowest segment of the pharynx had the highest values in patients with Angle class III. The volume of the oropharynx was found to be greater in patients with Angle class III versus patients with Angle class II.

## 1. Introduction

The link between respiratory function and craniofacial morphology has been a topic of interest in recent years. Previous research has shown the connection between upper airway and stomatognathic development [1,2,3,4,5,6]. As a result of upper airway restriction or obstruction, changes in breathing may occur, directly influencing normal craniofacial development and dental positions [1]. The ideal upper airway involves nasal breathing [7,8,9,10]. Normal nasal breathing involves air circulation through filtration [11,12]. Studies demonstrate multiple advantages of nasal breathing [13]. These functions can be compromised when upper airway obstruction occurs. Obstruction can be correlated with the functional activity and volume of the surrounding soft tissues and has been shown to be frequently encountered from an early age [14,15,16].

Mouth breathing can negatively affect growth and development, predisposing the subject to a lowered position of the mandible and tongue and changing the growth direction of the facial structures [15]. It may determine a clockwise rotation of the mandible and a vertical type of growth [17]. Depending on the position of the tongue and its action on the floor of the mouth, two types of anomalies can develop: Angle class II, with the tongue in a lower and posterior position, and Angle class III, with the tongue in a lower and anterior position [17,18,19,20,21]. An enlarged lower floor, dental crowding, difficulties in swallowing and mastication, the upper incisors proclinated and lower ones lingualized, and a deep palatal vault are also mentioned as complications of the altered tongue position [8,17]. Today, radiography is an indispensable tool in orthodontic practice. Studies show that more than a quarter of radiographs in the European Union are made in dental medicine [9]. However, two-dimensional (2D) imaging techniques have proven to be ineffective in reproducing three-dimensional (3D) structures and associated pathologies [9,22,23,24,25]. With the appearance of CBCT in the late 1990s, dental radiography was revolutionized [10]. CBCT has the great advantage of reconstructing inaccessible images from previous orthodontic practice, allowing the orthodontist to analyze multiple planes, such as axial, sagittal, and coronal. Studying tissues, facial soft structures, and dentition from infinite incidences is also possible. The second significant advantage is the possibility of extracting conventional radiographs, such as panoramic and lateral cephalograms, from a single CBCT scan [11,25,26,27]. Some studies [17,18,19] have investigated the correlation between the morphology of the upper airways and different growth patterns in sagittal and vertical planes. Di Carlo et al. studied [19] the upper airway’s morphology and dimensions through 3D radiological measurements in 90 young adult patients. The sagittal plane was assessed, and the patients were divided into three groups according to the value of the ANB angle. In their recent study, Sfondrini et al. [17] evaluated upper airways in adult Caucasian subjects without previous orthodontic treatment. Their main objective was to measure the upper airway dimensions in adult skeletal class I, II, and III patients [17]. However, their measurements were performed two-dimensionally in lateral radiographs [17]. The morphology of the pharyngeal airway in growing and nongrowing cleft lip and palate patients was assessed in Abdelkarim et al.’s study [22]. Their sample consisted of 36 cleft lip and palate subjects and 30 subjects without cleft lip and palate in the control group [22]. A meta-analysis [20] in 2022 aimed to evaluate scientific evidence related to the effects of different orthodontic treatment possibilities on the airways. The authors included in their meta-analysis 66 eligible articles about the CT and CBCT airway evaluation after orthodontic therapy [20]. The orthodontist has a vital role in recognizing a patient’s respiratory problem and correlating it with the type of malocclusion to prescribe the correct treatment.

The primary purpose of this study was to evaluate the following parameters: upper airway morphology and volume in class I, II, and III Angles. The secondary objective was to compare and correlate these parameters.

## 2. Materials and Methods

### 2.1. Study Design, Participants, Measurements, and Variables

The present retrospective observational study was approved by the Ethical Committee of the “George Emil Palade” University of Medicine, Pharmacy, Science, and Technology of Targu Mures (approval no. 2904/8 March 2024).

The sample size for this study was determined using G*Power version 3.1.9.6 software (Franz Faul, Universität Kiel, Kiel, Germany). The calculations indicated that a minimum of 16 patients per group (total sample size of 42) would be necessary; this size would provide greater than 95% power to detect significant differences, with an effect size of 0.80 at a significance level of α = 0.05. Thus, 90 preorthodontic CBCT scans of orthodontic patients (38 males, 52 females, mean age 42.29, SD = 10.23) were included in this study.

Based on the cephalometric values, the skeletal diagnosis of each patient was established: class I (ANB = 0–4°), 29 patients (11 males, 18 females, mean age 45.38); class II (ANB > 4°), 33 patients (15 males, 18 females, mean age 40.67); and class III (ANB < 0°), 28 patients (11 males, 16 females, mean age 41.00).

Each patient signed an informed consent agreeing to the use of these records. The following inclusion criteria were used: no previous orthodontic treatment, no surgical interventions on the upper/lower airway, no general illness with respiratory symptoms, age between 25 and 64 years. CBCTs were performed as part of the diagnostic process, thus ensuring that this study abides by good clinical practice. CBCTs (FOV 13 × 15) were performed using a KaVo OP 3D machine (Kavo LTD., Charlotte, NC, USA).

The skeletal sagittal patterns (ANB angle) were established using Steiner cephalometric analysis, an essential tool in orthodontic assessment and treatment planning, on the CBCT cross section representing the lateral cephalometric image. The images were uploaded using digital cephalometric software Romexis 3.6.0 (Romexis, Planmeca, Helsinki, Finland). The landmarks, angles, and planes for the cephalometric tracing were as follows:

- Nasion (N): most anterior point on the frontonasal suture in the midsagittal plane.
- Sella (S): center of the pituitary fossa of the sphenoid bone.
- Point A (A): deepest point of the curve of the anterior border of the maxilla.
- Point B (B): most posterior point in the concavity along the anterior border of the symphysis.
- SNA: angle between the Sella, Nasion, and A point; this angle measures the position of the upper jaw relative to the base of the skull.
- SNB: angle between the Sella, Nasion, and B point; this angle measures the lower jaw’s position relative to the skull’s base.
- ANB: angle between point A, the Nasion, and point B, indicating the relationship between the upper and lower jaws.
- SN: plane between the Sella and Nasion.
- Anterior nasal spine (ANS): anterior tip of the sharp bony process of the maxilla at the lower margin of the anterior nasal aperture.
- Posterior nasal spine (PNS): posterior limit of the palatine bone.

Based on the cephalometric values, the skeletal diagnosis of each patient was established: class I (ANB = 0–4°), class II (ANB > 4°), and class III (ANB < 0°). 

For the CBCT measurements, Hounsfield units were set between −1000 and −350. By setting the Hounsfield unit window to −1000 to −350, the CBCT images may be optimized to highlight structures such as bone, teeth, and soft tissues within the desired radiodensity range while minimizing interference from air or other low-density materials that fall below −1000 HU. Each case was analyzed in several sections within the 3D OnDemand program, especially the sagittal and axial sections.

The anatomic landmarks and planes related to upper airway analysis are presented in Table 1.

**Figure 1:**
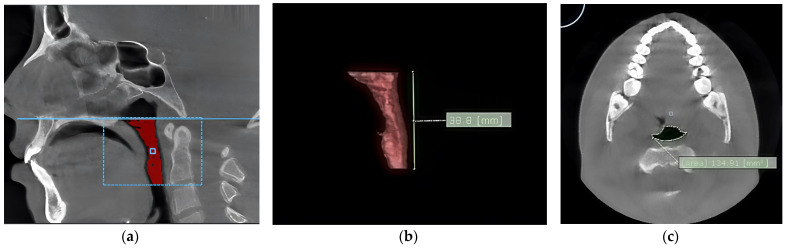
The aspect of the oropharynx. (**a**) The volume of the oropharynx; (**b**) the length of the oropharynx; (**c**) the area of the narrowest segment of the pharynx.

The volume of the pharynx (V-PA) was divided into the volume of the oropharynx (V-OA) and the volume of the hypopharynx (V-HA), which were summed up, resulting in the volume of the pharynx (V-PA) (Figure 1a). The upper limit of the oropharynx was the posterior nasal spine, and the lower limit was the antero-inferior border of the C2 vertebra. The upper limit of the hypopharynx was the antero-inferior border of the C2 vertebra, and the lower limit was the superior border of the hyoid bone. In addition, for calculating the volumes, the vertical length of the oropharynx (L-OA) and the vertical length of the hypopharynx (L-HA) were also calculated, which were summed up, generating the vertical length of the pharynx (L-PA) (Figure 1b).

After measuring the volumes of interest, editing tool functions were used to eliminate the unwanted hollow structures. The methodology used was similar to the one described by Meehan [28]: the interest zone was threshold-segmented, and the slice was edited by hand to remove any artifacts. After segmentation, the software automatically computed pharyngeal airway volumes in cubic millimeters, and the cross-sectional area (in square millimeters) was displayed on the axial image.

The area of the narrowest part of the pharynx (MIN-CSA) was also calculated (Figure 1c). Scrolling through all cross-sectional images determined the most constricted cross-sectional area (Min-CSA) of the pharynx. The vertical length of the Min-CSA (L-CSA) was measured with linear measurement tools. It was defined as the distance between the upper border of the oropharynx and the Min-CSA in the midsagittal view. The volume calculation was performed in cubic millimeters (mm^3^).

### 2.2. Data Measurement 

Two experienced orthodontists performed the cephalometric analysis. After two weeks, reliability was assessed by reperforming 50% of the cephalometric analyses.

### 2.3. Statistical Methods

The recorded values were analyzed statistically. The statistical analysis was performed using GraphPad Prism 8 for macOS version 10.2.1 (GraphPad Software, Boston, MA, USA). The mean (M), median (Me), and standard deviation (SD) were calculated. The statistical significance was set at *p* < 0.05. Given the non-normal distribution of the data, as confirmed by the Shapiro–Wilk test, and the heterogeneity of variances indicated by Levene’s test, we employed the Kruskal–Wallis test for the initial analysis. This choice was guided by the test’s suitability for nonparametric data. After identifying significant differences, Dunn’s post hoc test was proposed to pinpoint specific group disparities. The Mann–Whitney U test explored gender differences in pharyngeal volume and morphology among class I, II, and III individuals.

## 3. Results

The Kruskal–Wallis test revealed significant differences in the distribution of the V-OA (*p* = 0.0429) and V-PA (*p* = 0.0259) across the classes, suggesting variability in these parameters’ behaviors (Table 2). Conversely, the V-HA did not exhibit statistically significant differences (*p* = 0.4015), indicating similar distributions across the groups. Significant differences were found across the classes regarding the MIN-CS (*p* = 0.0004). A significant difference was also found in the distribution of the L-OA across the classes (*p* = 0.0180). No statistical difference was found between the L-HA and L-PA across classes.

**Figure 2:**
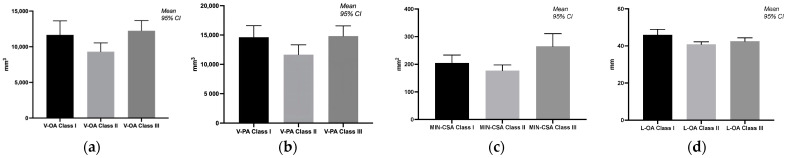
Graphical representation of the significant differences in the volume of pharyngeal airway spaces in different malocclusion classes. (**a**) Differences between the volume of the oropharynx in different malocclusions; (**b**) differences between the volume of the pharynx in different malocclusions; (**c**) differences between the areas of the narrowest part of the pharynx; (**d**) differences in the vertical length of the oropharynx in different malocclusions.

A significant change in the volume of the oropharynx and pharynx was demonstrated when comparing class II with class III anomalies (*p* = 0.0414, respectively, *p* = 0.0313). The total volume of the pharynx was increased in patients with class III anomalies. The area of the narrowest part of the pharynx (MIN-CSA) significantly decreased in classes I and II compared to class III (*p* = 0.0289, *p* = 0.0003). Between classes I and II, one parameter was modified, L-OA, with a *p*-value (*p* = 0.0138) being statistically significant (Figure 2).

**Figure 3:**
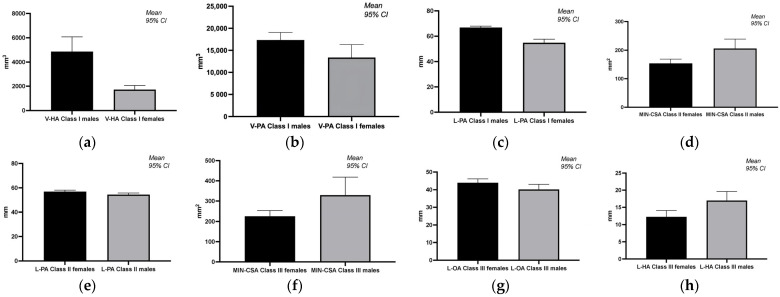
Graphical representation of significant differences in pharyngeal airway volumes and morphologies across different malocclusion classes and genders. (**a**) Gender differences in volume of the hypopharynx in class I; (**b**) gender differences in volume of the pharynx in class I; (**c**) gender differences in the sum of vertical length of oropharynx and hypopharynx in class I; (**d**) gender differences in the narrowest part of the pharynx in class II; (**e**) gender differences in the sum of vertical length of oropharynx and hypopharynx in class II; (**f**) gender differences in the narrowest part of the pharynx in class III; (**g**) gender differences in the vertical length of oropharynx in class III; (**h**) gender differences in the vertical length of hypopharynx in class III.

The statistical analysis (Mann–Whitney U test) highlights significant gender differences in pharyngeal volumes and morphologies across different malocclusion classes. Notably, males consistently displayed larger volumes and areas in specific measurements (e.g., V-HA, V-PA, and L-PA in class I; MIN-CSA and L-PA in class II; L-HA and MIN-CSA in class III), suggesting a predisposition towards larger pharyngeal dimensions compared to females (Table 3, Figure 3).

## 4. Discussion

In several studies, upper airway volumes were observed to differ by skeletal class [1]. Other studies demonstrate the correlation between class II and obstructive sleep apnea [18]. Regarding the delimitation of the segments, we used the same anatomical landmarks as those used by Zheng et al. [1]. Various studies have explored the relationship between malocclusion type (class I, class II, and class III), gender, and airway volume, revealing intricate interactions among these variables. Nath et al. [8] focused on the skeletal malocclusion’s influence on oropharyngeal airway volume using cone beam computed tomography (CBCT) analysis. This research found significant differences in airway volume among patients with different classes of skeletal malocclusion, underscoring the utility of CBCT in airway assessment [8]. Our results showed significant differences in the volume and area measurements of the oropharynx and pharynx when comparing different classes of anomalies (class II and class III) in orthodontic patients. The total volume of the pharynx was increased in patients with class III anomalies. The area of the narrowest part of the pharynx (MIN-CSA) was significantly decreased in classes I and II when compared to class III (Table 2, Figure 2). Rivlin et al. [18] demonstrated that an anterior mandible position in patients with Angle class III leads to an increased pharyngeal space. In patients with Angle class II, the mandible adopts a retrognathic position, and pharyngeal space proves to be significantly reduced [18]. Other researchers observed a difference in patients with Angle class III, where the hypopharynx volume was higher than in patients with Angle class II [1,28]. Understanding these differences can affect treatment planning and outcomes in orthodontic patients with different malocclusions. A CBCT-based comparison of pharyngeal airway area and volume in patients with Angle class I and class II malocclusions revealed that male patients had a greater area than female patients. However, no association was found between Angle classes I or II malocclusions and oropharyngeal airway volume [29]. A study by Kim et al. [30] highlighted gender-specific differences in airway dimensions among smokers, demonstrating that women have higher wall-area percentages and lower luminal areas, internal diameters, and airway thicknesses than men in anatomically matched airways. These findings highlight the influence of gender on airway dimensions [30]. According to Dominelli et al. [31], healthy women have central airways significantly smaller (~26–35%) than men, with the trachea showing the most significant difference. This difference persists even when subjects are matched for height [31]. Gungor and Turkkahraman reviewed the effects of airway problems on maxillary growth, highlighting the role of specific dental and skeletal malocclusions in airway volume differences [32]. S. Kim et al., in their study about identifying optimal oropharyngeal airway sizes for men and women, found nuanced differences in airway management between genders [33]. These differences affect clinical practices, such as airway management and assessing respiratory health risks. Some studies [34,35] about the association between gender, malocclusion, and the volume of the hypopharynx provide insights into the morphological characteristics of the hypopharynx and its variations by gender. A study by Zhang et al. [34] highlighted morphological characteristics of the male and female hypopharynx through MRI imaging. It was observed that female subjects had a smaller laryngeal cavity and piriform fossa compared to males, indicating gender differences in the morphology of the hypopharynx [34]. According to our study, males have a predisposition towards larger pharyngeal dimensions compared to females across the different malocclusion classes. Male orthodontic patients presented higher volumes and areas in measurements (V-HA, V-PA, and L-PA values for class I; MIN-CSA and L-PA in class II; and MIN-CSA and L-HA in class III). While specific studies directly associating malocclusion with the volume of the hypopharynx were not found, research into the general effects of malocclusion on oral health indicates potential pathways through which malocclusion could impact the hypopharyngeal region. For instance, Rantavuori et al. observed gender differences in the association between malocclusion traits and oral health-related quality of life in Finnish adults, suggesting that malocclusion can have systemic effects that might extend to the hypopharyngeal area in terms of overall health and quality of life [35]. A study focusing on patients with catathrenia, a sleep-related breathing disorder, found that these patients had a statistically smaller sagittal diameter of the hypopharynx than the standard reference [36]. This finding, presented by Yu et al., indicates an association between malocclusion (as an indirect factor through conditions such as catathrenia) and a narrow hypopharynx. However, it does not directly address the role of gender [36]. The interplay between gender, malocclusion, and hypopharynx volume remains under-researched. The available studies primarily focus on gender differences in hypopharynx morphology or the broader impacts of malocclusion. Direct studies exploring all three variables are scarce, highlighting an area for future research. According to our study, malocclusion type and gender significantly influence airway volume. The differences resulting from the present study follow the findings from the literature [8,29,37] and have important implications for the assessment and treatment planning in individuals with malocclusion. They highlight the need for personalized approaches for dental occlusion types and gender-specific airway characteristics.

From a clinical point of view, the findings of our study are relevant when orthodontic treatment is initiated in patients with different classes of anomalies. In the case of class II anomalies, treatment options that might reduce the volume of the pharynx should be avoided. The use of digital dentistry might enhance the outcome of the treatment [19,38,39,40,41,42,43,44]. 

The limitations of the present study are the following. The evaluation of the airways was performed only in sagittal malocclusion. This study is retrospective. The unregulated respiratory cycle during image acquisition and inadvertent variations in tongue positioning during CBCT scans could affect the accuracy of static 3D images. We lacked control over variables like head position, tongue position, and breathing during CBCT scans. While cone beam computed tomography (CBCT) offers detailed images and has revolutionized dental radiography, it still has limitations. These include the potential for distortion in the imaging of airway structures and the reliance on the proper setting of Hounsfield units for optimal visualization. The interpretation of these images may also be subject to operator error or variability. This study divides participants into three skeletal classes based on ANB angle measurements. However, it does not mention the distribution of other potentially influential factors, such as ethnicity or body mass index. This study provides a snapshot in time and does not track changes throughout orthodontic treatment or as participants age. Therefore, it cannot establish causality or assess the long-term implications of the observed skeletal patterns on airway volume.

Future research could consider replicating the analysis while incorporating sagittal discrepancies, other malocclusions, and functional alterations. By deepening the understanding of airway alterations related to maxillofacial morphology, researchers can more effectively detect patients who are at risk of airway dysfunction early on. Further investigations and analysis after finishing the orthodontic correction of different malocclusions may help elucidate the clinical significance of these anatomical variations and their relationship to orthodontic treatment outcomes. However, future research should focus on prospective studies with larger sample sizes and standardized protocols to further validate these findings and enhance treatment strategies for patients with orthodontic and airway concerns. Also, the analysis can be extended to include the role of surrounding soft tissues, such as tonsil size and tongue posture, in affecting airway volume and orthodontic treatment outcomes. Gender-specific research to delve deeper into the observed gender differences in pharyngeal volumes and morphologies, potentially including hormonal influences, is also necessary.

## 5. Conclusions

Within the limitations of the present study, the narrowest segment of the pharynx had the highest values in patients with Angle class III. The volume of the oropharynx was found to be greater in patients with Angle class III versus patients with Angle class II. 

The findings from this study underscore the importance of considering gender when evaluating pharyngeal volumes and areas in orthodontic patients with malocclusions. Understanding these differences is crucial for the diagnosis and treatment planning of malocclusions, potentially impacting approaches to managing airway-related issues in orthodontic patients.

## Figures and Tables

**Table 1:** Anatomic landmarks of the upper airways.

	Superior Limit	Inferior Limit
Oropharynx	Line extending from the PNS to the tip of the odontoid process.	Line extending from the antero-inferior border of the C2 vertebra.
Hypopharynx	Line extending from the antero-inferior border of the C2 vertebra.	Line extending from the horizontal line coming into contact with the most superior margin of the body of the hyoid bone.

**Table 2:** Pharyngeal airway spaces in the skeletal patterns using the Kruskal–Wallis test with Dunn’s post hoc test.

Variables	Class I		Class II		Class III		Intergroup Comparison *
	Mean	SD	Mean	SD	Mean	SD	
V-OA	11,660	5076	9313	3156	12,256	3647	-
V-HA	2966	1979	2354	1655	2567	1151	class III > class I > class II
V-PA	14,626	5094	11,667	4280	14,823	4479	-
MIN-CSA	204.9	74.56	177.2	52.39	264.8	117.9	class III > class I > class II
L-OA	45.99	7.603	40.92	3.468	42.60	4.589	class III > class I > class II
L-HA	13.23	5.911	14.62	4.318	14.39	4.480	class I > class III > class II
L-PA	59.22	7.628	55.60	2.746	56.99	4.718	-

* Significant differences.

**Table 3:** Gender differences in pharyngeal volumes and morphologies across different malocclusion classes using the Mann–Whitney U test.

Variables	Class I			Class II			Class III		
	Male	Female	*p*-Value	Male	Female	*p*-Value	Male	Female	*p*-Value
	Mean (SD)	Mean (SD)		Mean (SD)	Mean (SD)		Mean (SD)	Mean (SD)	
V-OA	12,484 (4256)	11,671 (5907)	0.5397	10,245 (2532)	8673 (3717)	0.1152	12,491 (3222)	12,443 (3374)	0.9746
V-HA	4857 (1823)	1711 (684.5)	0.0003 ***	3127 (2121)	2049 (1171)	0.0559	3095 (1145)	2278 (972.3)	0.4506
V-PA	17,341 (2546)	13,383 (5856)	0.0069 **	13372 (3995)	10,722 (4557)	0.2135	15,587 (4234)	14,721 (3930)	0.5248
MIN-CSA	198.4 (41.62)	213.2 (89.79)	0.8605	206 (59.8)	153.9 (29.94)	0.0091 **	329.4 (140.3)	225.5 (51.65)	0.0184 *
L-OA	49.74 (6.171)	44.24 (7.915)	0.0834	39.1 (3.207)	42.3 (3.303)	0.0559	40.19 (4.595)	43.88 (4.202)	0.0118 *
L-HA	17.07 (7.392)	10.58 (2.844)	0.0316 *	15.2 (4.115)	14.55 (4.599)	>0.9999	16.98 (4.179)	12.28 (3.404)	0.0043 **
L-PA	66.81 (1.679)	54.82 (5.839)	<0.0001 ****	54.42 (2.416)	56.85 (2.496)	0.0221 *	57.17 (6.653)	56.15 (1.548)	0.7547

* Significant, ** very significant, ***, highly significant, **** extremely significant.

## Data Availability

The data presented in this study are available on request from the corresponding author.
